# Measuring the True Costs of War: Consensus and Controversy

**DOI:** 10.1371/journal.pmed.1000417

**Published:** 2011-02-15

**Authors:** Robert Muggah

**Affiliations:** Graduate Institute of International and Development Studies, Geneva, Switzerland

## Abstract

Robert Muggah discusses the costs of war and a new analysis published in *PLoS Medicine* by Madelyn Hsiao-Rei Hicks and colleagues that documents the number of Iraqi civilian violent deaths during 2003-2008.

**Linked Research Article**

This Perspective discusses the following new study published in *PLoS Medicine*:

Hicks MH-R, Dardagan H, Guerrero Serdán G, Bagnall PM, Sloboda JA, et al. (2011) Violent Deaths of Iraqi Civilians, 2003–2008: Analysis by Perpetrator, Weapon, Time, and Location. PLoS Med 8(2): e1000415. doi:10.1371/journal.pmed.1000415

Madelyn Hsiao-Rei Hicks and colleagues provide a detailed analysis of the Iraqi civilian violent deaths that occurred during 2003–2008 of the Iraq war, and show that of 92,614 deaths, unknown perpetrators caused 74% of deaths, Coalition forces 12%, and Anti-Coalition forces 11%.

All wars generate controversy, though some more so than others. The current US-led intervention in Iraq and the catastrophic explosion of ethnic violence it incited is especially contentious. Whilst acrimonious disagreement over the *raison d'etre* of the Iraq war persists in capitals around the world, few deny that it has been an especially bloody and traumatic experience for Iraqi civilians. The full extent of suffering is confirmed in this week's *PLoS Medicine*, which features an article by Madelyn Hicks and colleagues who consider the human costs of Iraq's war by focusing on civilian deaths between 2003 and 2008 [1].

While offering fresh new insights on the nature and extent of violence in the country, this is not the first article that has considered deaths arising from the Iraqi conflict [2]–[4]. Nor is it likely to be the last. Rather, it should be set alongside a growing cannon of work originating from public health sciences and conflict studies devoted to examining the human consequences of the Iraq war. But the focus and time period investigated by Hicks and colleagues is significant: they track the characteristics of 92,000 direct civilian deaths occurring during the start, escalation, and dramatic reduction of war-related violence. In this respect, their article builds on and goes beyond assessments undertaken in the recent past.

## The Costs of War

Though armed conflict has persisted for tens of thousands of years, preoccupation with the costs of war in terms of human pain and suffering is a phenomenon of the modern era. The first truly international response to the human suffering generated by warfare emerged following the Battle of Solferino around the middle of the 19th century, when more than 160,000 Austrian troops did battle with 156,000 French and allied forces on Italian soil. Horrified by the savagery inflicted by combatants on one another, a Swiss banker, Jean-Henri Dunant, initiated a process that led to the Geneva Conventions and the establishment of what eventually became known as the International Red Cross and Red Crescent movement.

Public awareness of the impacts of warfare on human wellbeing expanded during the massive international and internal wars of the 20th century. There was mounting concern not just for soldiers wounded and dying in battle, but also for the devastating implications of large-scale industrial warfare on civilians. Until at least the 1950s and 1960s, the aerial bombardment and often indiscriminate targeting of citizens was treated by leaders as a regrettable, but in some cases unavoidable, form of collateral damage. A remarkable shift in attitude followed the end of Cold War, owing in part to the growing influence of the global media in shaping the understanding of and responses to the world's war-affected hot spots.

Today there are considerable awareness and consensus about the causes and consequences of armed conflict in the roughly two dozen countries presently affected. Since the 1990s a veritable industry has developed, dedicated to measuring and monitoring the casualties brought about by civil war. There are literally dozens of research centers and initiatives littered throughout North and South America, Western Europe, and Australia devoted to the task of tracking the incidence of mortality and morbidity, many of them applying broadly similar methods. Most scholars now agree that while most 21st century wars are nasty and brutish, they are in fact declining in number and intensity [5].

## The Controversies about Counting Methods

Notwithstanding widespread commitment among health and conflict specialists to bear witness and make public the real costs of war, there is comparatively less consensus about how such accounting ought to be pursued. There are in fact fundamental disagreements about the most appropriate methodological approach to counting “conflict deaths,” whether amongst soldiers or civilians. Scholars involved in collecting data and estimating the incidence of violent death rapidly divided into two camps: the *incident reporters* and the *survey administrators*. Disagreements between these two groups are by no means trivial—they have profound implications on how the pathways of armed conflict are assessed and the scale and distribution of their impact are determined, and on the way solutions are constructed and implemented.

Very generally, most social scientists favor incident reporting that documents the number of people dying as a result of warfare on the basis of official data, authoritative media reports, independent studies, morgues, hospitals, clinics, and a range of available non-governmental agency sources. Although incident reporters are keenly aware of the selection biases that can be introduced into their analysis, the distortions generated by missing data, and the potential for censoring, they nevertheless contend that incident reporting offers critical insights. Trained in both the health and social sciences, Hicks and her co-authors are devoted proponents of this approach. Alongside their work are recent innovations such as the Armed Conflict Location and Events Dataset (ACLED), which reveals geospatial and temporal patterns of violence as well as situational analysis of events and vectors [6].

On the other side are epidemiologists and public health experts who favor the use of probabilistic sampling and survey-based approaches. Recognizing that in most countries vital registration data are weak and incident reporting uneven, survey administrators routinely invest in retrospective and prospective surveys to assess all manner of vulnerabilities, including the risk or fact of death and injury. The approach typically involves the randomized sampling of households in conflict zones in order to obtain basic data on family size, adult and child mortality rates, and causes of death. A number of such surveys have been undertaken in Iraq since 2003 [7]–[9]. The World Health Organization (WHO), along with projects such as the standardized monitoring and assessment of relief and transition (SMART) and complex emergency database (CE-DAT) are exponents of this latter approach.

Since the onset of the Afghanistan and Iraq wars, the debate on the human costs of armed conflict have become more heated [10]–[13]. Researchers have succeeded in drawing considerable public attention to the ways in which warfare yields devastating consequences, in both intended and unintended ways. Yet there are still some unresolved questions about whether war deaths are increasing or decreasing globally and over time [14],[15]. Equally intense disagreement persists between the incident reporters and survey monitors concerning the methodological validity of the others methods—particularly their underlying assumptions about baseline mortality rates, the nature and distribution of violence, sampling procedures, and latent biases in reporting [16],[17].

## Where We Stand in Iraq

The debates over how many people have been killed since 2003 in Iraq—and who did the killing—are of critical importance in setting the record straight. It is only through the generation of reliable and valid analysis that decision-makers and their armed forces can be held to account and that any form of meaningful lessons can be taken from such human-made disasters. Fortunately, Hicks and her colleagues extend the analysis beyond the numbers by applying a “dirty war index” (DWI) [18], which measures the proportion of women and children killed during hostilities. Their conclusions make for disturbing reading: while observing an escalation of extrajudicial killings and the use of mortars and vehicle bombs by unknown perpetrators, they attribute a high DWI to coalition and anti-coalition forces alike.

It is worth recalling that while counting the human toll of war in conflict zones such as Afghanistan, Colombia, the Democratic Republic of Congo, Iraq, and Sudan are of great importance, the true magnitude of the toll should not be underestimated. As survey administrators well know, the impacts of warfare extend well beyond the number of soldiers and civilians killed and injured on or near the battlefield. While it is extremely challenging to access insecure areas, public health specialists recognize that the vast proportion of mortality and morbidity arising from war occurs indirectly, owing to easily preventable diseases such as dysentery and measles, as well as malnutrition. Death rates are aggravated by the collapse of basic health infrastructure, adequate food and shelter, clean water, and other basic needs. Developing a full accounting of the costs of warfare, while difficult, is both an obligation and responsibility.

## Abbreviations

ACLED: Armed Conflict Location and Events Dataset
CE-DAT: complex emergency database
DWI: “dirty war index”
SMART: standardized monitoring and assessment of relief and transition
WHO: World Health Organization

## References

[1] Hicks MH-R, Dardagan H, Serdán C, Bagnall P, Sloboda S (2011). Violent deaths of Iraqi civilians over five years following 20 March 2003 by perpetrator, weapon, time and location.. PLoS Med.

[2] Dardagan H, Sloboda J, Williams K, Bagnall P (2005). Iraq body count: a dossier of civilian casulties 2003-2005..

[3] Daponte B (2007). Wartime estimates of Iraqi civilian casualties.. International Review of the Red Cross.

[4] Iraq Family Health and Survey Group (2008). Violence-related mortality in Iraq from 2002 to 2006.. N Engl J Med.

[5] Human Security Report Project (2010). http://www.hsrgroup.org/human-security-reports/20092010/overview.aspx.

[6] Armed Conflict Location and Events Dataset (2010). http://www.acleddata.com.

[7] Hicks M, Dardagan H, Guerrero Serdán G, Bagnall P, Sloboda J (2009). The weapons that kill civilians—deaths of children and noncombatants in Iraq, 2003-2008.. N Engl J Med.

[8] Burnham G, Lafta R, Doocy S, Roberts L (2006). Mortality after the 2003 invasion of Iraq: a cross-sectional cluster sample survey.. Lancet.

[9] Roberts L, Lafta R, Garfield R, Khudhairi Jl, Burnham G (2004). Mortality before and after the 2003 invasion of Iraq: cluster sample survey.. Lancet.

[10] Bohannon J (2006). Epidemiology. Iraqi death estimates called too high; methods faulted.. Science.

[11] WHO (2008). http://www.who.int/mediacentre/news/releases/2008/pr02/en/index.html.

[12] The Associated Press (28 October 2004) Scientists estimate that more than 100,000 Iraqis may have died during the war. USA Today. Available: http://www.usatoday.com/news/world/iraq/2004-10-28-casualties_x.htm. Accessed 12 January 2011

[13] Roberts L (14 February 2007). http://www.independent.co.uk/opinion/commentators/les-roberts-iraqs-death-toll-is-far-worse-than-our-leaders-admit-436291.html.

[14] Murray CJ, King G, Lopez AD, Tomijima N, Krug EG (2002). Armed conflict as a public health problem.. BMJ.

[15] Obermeyer Z, Murray C, Gakidou E (2008). Fifty years of violent war deaths from Vietnam to Bosnia: analysis of data from the World Health Survey Programme.. BMJ.

[16] Spagat M, Mack A, Cooper T, Kreutz J (2009). Estimating war deaths: an arena of contestation.. Journal of Conflict Resolution.

[17] Wikipedia (2010). http://en.wikipedia.org/wiki/Lancet_surveys_of_Iraq_War_casualties#cite_note-Bohannon-66.

[18] Hicks MH-R, Spagat M (2008). The Dirty War Index: a public health and human rights tool for examining and monitoring armed conflict outcomes.. PLoS Med.

